# Resistome-based surveillance identifies ESKAPE pathogens as the predominant gram-negative organisms circulating in veterinary hospitals

**DOI:** 10.3389/fmicb.2023.1252216

**Published:** 2023-09-07

**Authors:** Flavia Zendri, Cajsa M. Isgren, Jane Devaney, Vanessa Schmidt, Rachel Rankin, Dorina Timofte

**Affiliations:** ^1^Department of Veterinary Anatomy, Physiology and Pathology, Institute of Infection, Veterinary and Ecological Sciences, University of Liverpool, Neston, United Kingdom; ^2^Western Counties Equine Hospital Ltd., Culmstock, United Kingdom; ^3^Department of Equine Clinical Science, Institute of Infection, Veterinary and Ecological Sciences, University of Liverpool, Leahurst Campus, Neston, United Kingdom; ^4^Department of Small Animal Clinical Science, Institute of Infection, Veterinary and Ecological Sciences, University of Liverpool, Leahurst Campus, Neston, United Kingdom

**Keywords:** veterinary, infection control, gram-negative, ESKAPE, companion animals, surveillance, veterinary hospitals, intensive care unit (ICU)

## Abstract

**Introduction:**

Healthcare-associated infections (HCAIs) associated with extended-spectrum cephalosporin-resistant gram-negative (ESC-R GN) bacteria are an emerging concern in veterinary hospitals, especially in companion animal intensive care units (ICUs).

**Methods:**

To understand the molecular epidemiology of ESC-R GN isolates in two veterinary hospitals (equine and small animal), a 6-month pilot study was performed during which fecal and environmental samples were obtained twice from selected patients, upon ICU admission and after 48 h of hospitalization. In total, 295 ESC-R GNs were analyzed using the Acuitas Resistome^®^ Test (OpGen, Maryland, US), a PCR-based assay screening for 50 antimicrobial resistance gene families encoding for production of extended-spectrum beta-lactamase (ESBLs), TEM/SHV/OXA or AmpC beta-lactamases and carbapenemases. Combining organism identification and antimicrobial susceptibility data to genotyping results, unique “Acuitas profiles” were generated that can be used for fast typing the isolates and tracking transmission events.

**Results:**

ESKAPE GN pathogens were the most prevalent ESC-R GN isolates circulating in both the small animal and equine hospitals, consisting of *Enterobacter cloacae* complex (21.7%), *Pseudomonas aeruginosa* (20%), *Klebsiella pneumoniae* (15.9%), and *Acinetobacter baumannii* complex (13.6%) followed by *Escherichia coli* (12.2%), most harboring a combination of genes encoding for beta-lactamases and ESBLs. Some ESKAPE genotypes showed likely intra-hospital transmission, including *E. cloacae* (two genotypes, one carrying SHV4, SHV5, and TEM7 and the other TEM1, TEM3, and TEM7 enzymes) in the equine and *K. pneumoniae* (SHV1, SHV5, and DHA1-positive) in the small animal ICUs, respectively. Furthermore, *P. aeruginosa* (carrying OXA-50), *A. baumannii* complex (OXA-51), and *E. coli* (CTX-M-1) genotypes were isolated across both hospitals, suggesting possible transfer mediated via movement of staff and students. Importantly, isolates carrying transmissible resistance to last-resort antimicrobials (i.e. carbapenems) were identified within the hospital environments, consisting of three environmental *Acinetobacter* spp. harboring *bla*_OXA − 23_ and one clinical *E. coli* with *bla*_OXA − 48_.

**Conclusion:**

We describe the widespread occurrence of ESKAPE gram-negative organisms in veterinary ICU patients and hospital environments. Findings from this project provide baseline data on the epidemiology of ESKAPE pathogens in veterinary settings, which can inform infection control policies to aid in patient management and prevent transmission of nosocomial infections associated with these pathogens.

## 1. Introduction

Nosocomial infections, also known as healthcare-associated infections (HCAIs), are either localized or systemic infections that are typically not present at the time of admission but are acquired by patients during their stay in a hospital or other healthcare facility and usually manifest approximately 48 h after admission to the hospital (Monegro et al., 2021). Approximately 4,100,00 new cases of HAI are estimated to occur every year in people in the European Union and European Economic Area (EU/EEA) with the number of deaths occurring as a direct consequence of these infections estimated to be at least 37,000.^1^ Of these, the number of HCAIs caused by antimicrobial-resistant (AMR) microorganisms was calculated to be 426,277 occurring in the EU every year (Cassini et al., 2019). In particular, multidrug-resistant (MDR) HCAIs are a major challenge for both human and veterinary medicine as they are associated with increased morbidity and mortality rates as well as increased healthcare costs. Importantly, gram-negative (GN) bacteria within the ESKAPE group of pathogens (***E****nterococcus faecium*, ***S****taphylococcus aureus*, ***K****lebsiella pneumoniae*, ***A****cinetobacter baumannii*, ***P****seudomonas aeruginosa*, and ***E****nterobacter* spp.) pose a real threat due to their tendency to become MDR and thereby “escape” most antimicrobial agents.

In contrast to human medicine, data on the occurrence of HCAIs in veterinary medicine remain limited although the problem has recently gained increasing awareness (Stull and Weese, 2015). In addition, infection control still remains in its infant stages despite animal HCAIs increasing importance in modern veterinary practice (Walther et al., 2017). This appears to be particularly the case for companion animals, i.e., dogs, cats, and horses, where a growing body of literature has described nosocomial outbreaks of different etiologies which are often associated with, and complicated by, the antimicrobial-resistant and zoonotic nature of the microorganisms involved (Walther et al., 2017). Therefore, their occurrence has great potential to hamper treatment, resulting in poor patient outcomes and extensive outbreaks that can affect not just hospitalized animal patients but also veterinary staff and animal owners.

Environmental contamination of veterinary hospitals and clinics may be an important source of subsequent infection (Murphy et al., 2010) as outlined by studies exploring the colonization burden of patients upon admission or during hospitalization (Gibson et al., 2011; Van den Eede et al., 2012) and studies investigating correlations between clinical infections and environmental detection of targeted pathogens (Weese et al., 2006; Timofte et al., 2016; Bortolami et al., 2017). One important component of nosocomial infection development is the widespread fecal (but also cutaneous or upper respiratory) carriage of multidrug-resistant gram-negative (MDR-GN) pathogens by animals entering veterinary facilities and/or by staff members, with potential direct or indirect dissemination to other patients and, conceivably, seeding, and persistence within the hospital environment (Royden et al., 2019; Soza-Ossandón et al., 2020). Hospital settings are ideal for the development and selection of MDR organisms due to high antibiotic use and selective pressure (Mulvey and Simor, 2009). The development of large and specialized veterinary hospitals providing high-standard animal care involving complex interventions and state-of-the-art intensive care facilities has created similar conditions for the emergence of MDR-GN organisms adapted to the veterinary hospital environment. Studies investigating the risk factors for animal patients becoming carriers of MDR agents (Damborg et al., 2012; Maddox et al., 2012) have shown that environmental contamination with nosocomial pathogens is an important reservoir for subsequent infection (Grönthal et al., 2014; Timofte et al., 2016). Unlike methicillin-resistant *Staphylococcus aureus* (MRSA), which is widely studied in animals and where there is plentiful information regarding shared clones in humans and animals (Harrison et al., 2014; Haenni et al., 2017; Islam et al., 2017), much less is known and understood about the prevalence and epidemiology of MDR-GN pathogens in veterinary hospital environments.

Thus, this study aimed to generate veterinary-specific data on the molecular epidemiology of extended-spectrum cephalosporin-resistant (ESC-R) GN bacteria in small animal and equine veterinary hospitals. Ideally, to be able to implement effective preventative measures, detection of HCAIs infections shall be performed in real time; for this reason, our data were generated by using a fast bacterial strain-typing tool (Acuitas^®^ Resistome) to investigate the introduction, transmission, and/or persistence patterns of MDR-GN bacteria within small animal and equine veterinary hospital settings, with emphasis on the intensive care units (ICUs).

## 2. Materials and methods

### 2.1. Study design

To understand the molecular epidemiology of ESC-R GN bacteria in veterinary environments, we performed a 6-month pilot study (PS) in the ICU of two veterinary referral hospitals (one equine and one small animal) at the University of Liverpool, England. Between January and June 2018, we aimed to recruit two to three patients/week admitted to the hospitals' ICUs. Fecal (F) and environmental (ENV) samples were collected as follows: freshly voided (horses) or passed (dogs) fecal samples were collected upon hospital admission (F1) and again after 48 h of hospitalization (F2) to determine whether intestinal carriage was community- or hospital-acquired. Environmental samples were collected at the same time points as for the fecal samples (ENV1 and ENV2) from high-touch surfaces surrounding the hospitalized patients. Specific bacterial culture protocols were followed to select ESC-R GN pathogens from these samples by using selective media. In addition, any ESC-R GN isolates obtained from clinical (PS-CL) and environmental (PS-ENV) specimens submitted for routine diagnostics from these hospitals during the same time frame were also included in the analysis.

Furthermore, retrospective clinical (RTS-CL) and environmental (RTS-ENV) ESC-R GN isolates obtained between March 2016 and December 2017 from the same hospitals through routine processing of clinical specimens or active hospital environmental surveillance were included in the downstream analysis. Ethics approval was obtained for patients enrolled in the study under the University of Liverpool's Ethical Committee (Reference number: VREC588).

### 2.2. Sample collection

#### 2.2.1. ICU pilot study

Between January and June 2018, selected equine and small animal patients admitted to the respective ICUs at the Liverpool Hospitals were enrolled. Informed consent was obtained from owners upon hospital admission. Paired fecal (F) and environmental (ENV) samples were collected on two separate occasions from each patient, specifically upon admission (F1 and ENV1 at *t0*) and after 48 h of hospitalization (F2 and ENV2 a *t1*). Approximately five grams (5 g) of fresh feces were collected in the early hospitalization hours by ICU staff and placed in sterile Universal containers before direct delivery to the on-site microbiology laboratory on the same day. Environmental ICU samples (*n* = 4 or 5 per patient at each timepoint) were collected at the same time of acquiring the fecal samples by trained veterinary technicians or nurses of the infection control team wearing gloves changed between samples. Environmental sites sampled consisted of animal and human high-contact surfaces; for small animals, a total of five ENV samples were collected that included the ICU telephone receiver and computer keyboard, the ICU door handle, the ICU floor, and the patient kennel (walls and floor). For horses, four ENV samples were submitted, consisting of combinations of the following: ICU door handle, water bucket, hay rack, feed bowl, tie ring, and pen window (ledge and bars). Environmental specimens were collected by swabbing ICU surfaces using sterile pre-moistened electrostatic Swiffer^®^ wipes approximatively 5 cm^2^ (Procter & Gamble, Ohio, US) to sample the entire object (e.g., for door handles, phone receiver) or a representative surface size (approximatively 0.5 m^2^ when possible, e.g., floor, walls). The sampling cloths were then folded and placed in bottles containing 250 ml of buffered peptone water (BPW). Clinical and environmental ESC-R GNs (PS-CL and PS-ENV) obtained during the pilot study from the ICU patients as well as the wider environmental hospital areas were also included in the analysis.

#### 2.2.2. Retrospective clinical and environmental samples

To depict the epidemiology of MDR-GN bacteria, retrospective (RTS) equine and small animal clinical (RTS-CL) obtained through routine diagnostics and environmental (RTS-ENV) GN isolates obtained from routine environmental surveillance between March 2016 and December 2017 were retrieved from the local bacterial strain collection and included in the analysis.

RTS-CL isolates originated from clinical specimens from both sterile and normally contaminated body sites and represented in most cases pure or mixed predominant cultures, respectively. These specimens consisted of infected skin and wound swabs including surgical site infections, urine collected by cystocentesis, feces, bile, orthopedic implants, cutaneous annexes, oropharyngeal swabs, blood, and abdominal fluid.

RTS-ENV isolates were obtained from the active surveillance programme which is an integral part of the local infection control strategy aimed to monitor the occurrence of MDR organisms in the hospital environment. Surfaces from high-risk areas such as surgical theaters, intensive care units, treatment areas, recovery boxes, equipment (endotracheal tubes, anesthetic equipment), and human high-contact surfaces (computer keyboards, door handles, phone receivers), which could represent “hot spots” for cross-contamination between the environment and the patients, between patients, and between patients and medical staff, are generally included in the routine surveillance on a rotating basis. Non-clinical areas are sometimes also included, such as the hospitals' receptions, pharmacies, washrooms, in-house laboratories, and staff and students' dedicated areas. Environmental specimens are collected by the infection control nurse, using the same method as described for the ENV sample collection in the pilot study above.

### 2.3. Laboratory processing of fecal, environmental, and clinical isolates in the ICU study and retrospective phase

#### 2.3.1. Fecal samples

To screen for ESC-R GNs, fecal samples (1–2 g) were inoculated into 20 ml BPW with overnight incubation at 37°C and sub-cultured onto eosin methylene blue agar (EMBA; Thermo Scientific) containing 1 μg/ml of cefotaxime (Sigma-Aldrich Ltd., UK) and incubated for 24 h at 37°C aerobically. All ESC-R isolates were sub-cultured onto 5% sheep blood agar (SBA, Oxoid, Basingstoke, UK) for bacterial identification.

#### 2.3.2. Environmental samples

For the environmental samples, targeted screening for ESC-R GNs was carried out beginning with an enrichment stage incubating the Swiffer in BPW at 37°C overnight, followed by sub-culture (10 μl) onto EMBA with cefotaxime (1 μg/ml) and Pseudomonas Selective Agar (all from Oxoid, Basingstoke, UK) incubated aerobically at 37°C for 18–24 h. If colonies were phenotypically different, each colony morphotype (including both EMBA positive and negative ones) was sub-cultured onto 5% SBA for bacterial identification.

#### 2.3.3. Clinical samples

Pilot (PS-CL) and retrospective clinical (RTS-CL) isolates were obtained through the Liverpool Veterinary Microbiology Diagnostic Service. Clinical specimens were processed according to the local diagnostic protocols for pathogen detection and antimicrobial susceptibility testing from different sample types, which in most cases included plating out on a non-selective media such as 5% SBA (Oxoid, Basingstoke, UK) and Fastidious Anaerobe Agar (FAA; E&O Laboratories Ltd., Bonnybridge, UK) cultured aerobically and anaerobically. Clinical isolates included in this study were selected based on their resistance to extended-spectrum cephalosporins, determined as part of routine antimicrobial susceptibility testing for clinical isolates. Cefpodoxime (10 μg) was used as the screening agent and testing was performed on Mueller-Hinton agar (MHA) according to the Clinical and Laboratory Standards Institute (CLSI) guidelines for processing and interpretation (CLSI, 2018a).

#### 2.3.4. Bacterial species identification

All clinical and environmental isolates obtained before 2018 were identified using the Analytical Profile Index (API) system and APIWEB Software (bioMérieux, Marcy-l'Étoile, France), and their identification was later confirmed via matrix-assisted laser desorption/ionization time-of-flight mass spectrometry (MALDI-TOF MS). Isolates acquired from 2018 onwards were directly identified by MALDI-TOF MS (MALDI Biotyper 4.1.100 Software, Bruker Daltonics, Bremen, Germany) with a score >2.0.

Following identification, the isolates selected for this study (PS and RTS) were batched, collected using Amies gel-based charcoal swabs, and sent to the OpGen Clinical Services Laboratory in the United States for the performance of the OpGen Acuitas^®^ Resistome Test (OpGen Inc., Gaithersburg, MD).

### 2.4. Acuitas resistome

The Acuitas^®^ Resistome Test can detect a large number of antimicrobial resistance genes in GN bacteria in a single run, providing comprehensive and rapid phenotypic/genotypic typing results. The methodology consists of two tests run in parallel: (i) the Acuitas Test screening for antibiotic resistance determinants and (ii) the MDR-GN culture screen with species identification and antimicrobial susceptibility testing (ID/AST) by VITEK2 (bioMérieux, Durham, NC). The Acuitas Test is a real-time polymerase chain reaction (qPCR) microfluidic array assay which screens for 50 antibiotic resistance beta-lactamase gene families, including those encoding production of extended-spectrum beta-lactamases (ESBLs) (CTX-M, TEM, SHV, BEL, BES, TLA, PER, VEB, GES, OXA-2, OXA-10, OXA-18), AmpC beta-lactamases (ACC, ACT, CMY, DHA, FOX, MIR, MOX), carbapenemases (GIM, IMI, IMP, NDM, SIM, SPM, VIM, KPC, SFC, NMC-A, SME, OXA-23, OXA-24, OXA-45, OXA-48, OXA-50, OXA-51, OXA-54, OXA-55, OXA-60, OXA-62), and non-ESBL beta-lactamases of the SHV/TEM/OXA types. The Acuitas Resistome Test qPCR methodology was illustrated by Reuben et al. (2017) and Voulgari et al. (2020). The antimicrobial panel comprised ampicillin/sulbactam, piperacillin/tazobactam, cefazolin, ceftriaxone, ceftazidime, cefepime, aztreonam, imipenem, meropenem, ertapenem, amikacin, gentamycin, tobramycin, ciprofloxacin, levofloxacin, trimethoprim/sulphomethoxazole, and tigecycline. The results were interpreted according to the CLSI human clinical breakpoints (CLSI, 2018b). Another important feature of the Acuitas Resistome is the ability to indicate possible strain relatedness of isolates. For this, phenotypic and genotypic results are combined to generate unique “Acuitas profiles” that can be used for typing, cluster identification and tracking transmission events, as clonal isolates share the same Acuitas profiles. Each profile comprises codes that identify (1) organism genus and species, (2) phenotype code determined by AST results, (3) listing of up to 3 AMR gene codes determined by the Acuitas Resistome Test, (4) a unique numerical code representing the pattern of all positive and negative assays from the Acuitas Resistome Test results, and (5) the AST profile code linked to the unique pattern of non-susceptible AST results (Supplementary Figure 1). Genetically similar types, which could represent related isolates, were established by combining the organism's name and the code for the Acuitas Resistome Test results. These types were further divided into subtypes using the profile's AST code. All results were uploaded on the Acuitas Lighthouse MDRO Management System (OpGen) website portal for real-time access.

## 3. Results

### 3.1. Samples and bacterial isolates from the ICU pilot study and retrospective phase

Overall, 279 samples (*n*= 49 fecal and *n*= 230 environmental) were collected during the ICU pilot study from 28 selected patients (equine, *n*= 20 and small animal, *n*= 8) admitted to the ICUs and their surroundings (Supplementary Table 1). The majority of samples originated from the equine hospital (199/279) and the remainder from the small animal hospital (80/279). In general, fecal samples were relatively equally distributed across the first (F1 = 26/49) and second (F2 = 23/49) sampling time points. However, F1 and/or F2 fecal samples could not be obtained for some patients, for which only one fecal sample at either time point was processed (Supplementary Table 1). Environmental ICU PS samples (*n* = 230, of which ENV1 = 120 and ENV2 = 110) comprised *n* = 160 equine ICU and *n* = 70 small animal ICU specimens. ENV2 sample sets could not be obtained for two patients (Supplementary Table 1). Additionally, clinical (PS-CL) and hospital environmental (PS-ENV) ESC-R GN isolates obtained from routine diagnostics during the same period were included amongst the PS isolates (Supplementary Table 2). In addition to the 28 patients enrolled in the pilot study, *n* = 42 ESC-R GN clinical isolates (PS-CL and RTS-CL) from diagnostic submissions of 36 other patients were included.

Table 1 summarizes all PS (*n* = 207) and RTS (*n* = 88) bacterial isolates included in this study (total of *n* = 295 ESC-R GNs).

**Table 1:** Summary of all pilot study (*PS*) and retrospective phase (*RTS*) companion animal ESC-R GN isolates included in the study (March 2016–June 2018).

**Small animal hospital**		**Equine hospital**	
***Pilot study (PS)***			
F1	2	F1	12
F2	3	F2	15
ENV1	26	ENV1	45
ENV2	15	ENV2	52
CL	24	CL	9
ENV	4	ENV	0
***Retrospective phase (RTS)***			
CL	6	CL	3
ENV	46	ENV	33

F1 and ENV1, fecal and environmental samples collected upon ICU admission; F2 and ENV2, fecal and environmental samples collected after 48 h from Intensive Care Unit (ICU) admission; CL, clinical isolates obtained from hospital routine diagnostic samples; and ENV, environmental isolates obtained from hospital routine environmental surveillance.

#### 3.1.1. Overall ESC-R gram-negative organisms' prevalence

**Figure 1:**
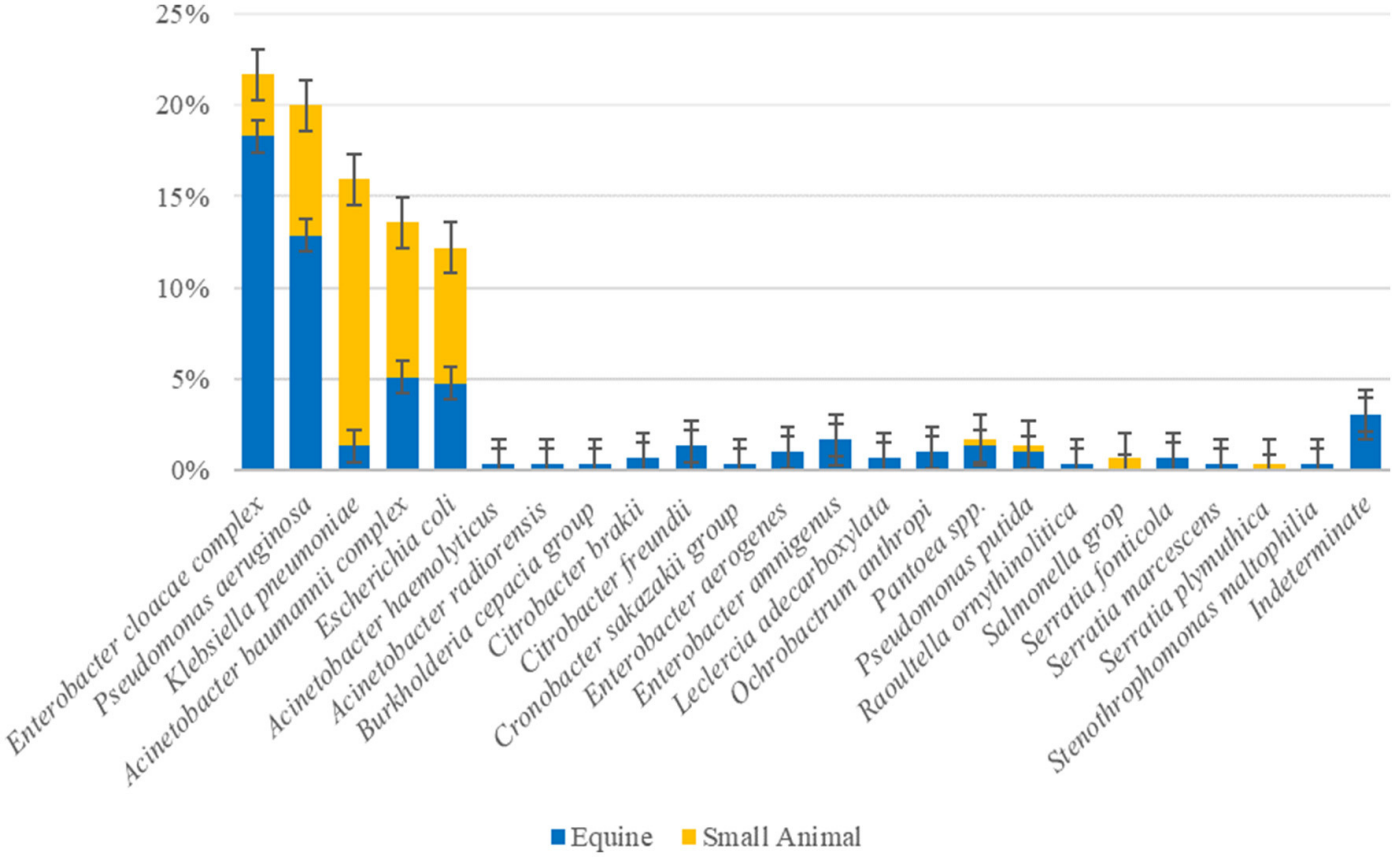
Overall prevalence of the ESC-R GN organisms (*n* = 295) circulating in the equine and small animal hospitals between March 2016 and June 2018. Error bars represent 95% confidence intervals.

The overall prevalence of companion animal ICU samples (F1&2 and ENV1&2) positive for ESC-R GNs during the pilot study (PS) was 51.3% (143/279) (Table 2). Twenty-three bacterial species were detected overall, with the most prevalent ESC-R GN organisms circulating in the equine and small animal hospitals between March 2016 and June 2018 being members of the ESKAPE group of pathogens, namely *Enterobacter cloacae complex* (64/295 = 21.7%), *Pseudomonas aeruginosa* (59/295 = 20.0%), *Klebsiella pneumoniae* (47/295 = 15.9%), and *Acinetobacter baumannii complex* (40/295 = 13.6%), followed by *Escherichia coli* (36/295 = 12.2%). Other organisms detected at significantly lower rates ( ≤ 5/295) are indicated in Figure 1.

**Table 2:** Prevalence data for samples collected from the Intensive Care Units (ICUs) during the pilot study (PS, January–June 2018).

**Small animal hospital**			**Equine hospital**				
**ICU pilot study**							
	**Samples**	**Positive**		**Samples**	**Positive**	**Tot samples**	**Tot positive**
F1	6	2	F1	20	11	26	13
F2	4	3	F2	19	13	23	16
ENV1	40	23	ENV1	80	35	120	58
ENV2	30	15	ENV2	80	42	110	57
Tot	80	52		199	100	279	143

Please note that PS-CL and PS-ENV isolates obtained through routine diagnostics during the same time frame were not included. F1 and ENV1, fecal and environmental samples collected upon ICU admission; F2 and ENV2, fecal and environmental samples collected after 48 h from ICU admission.

**Figure 2:**
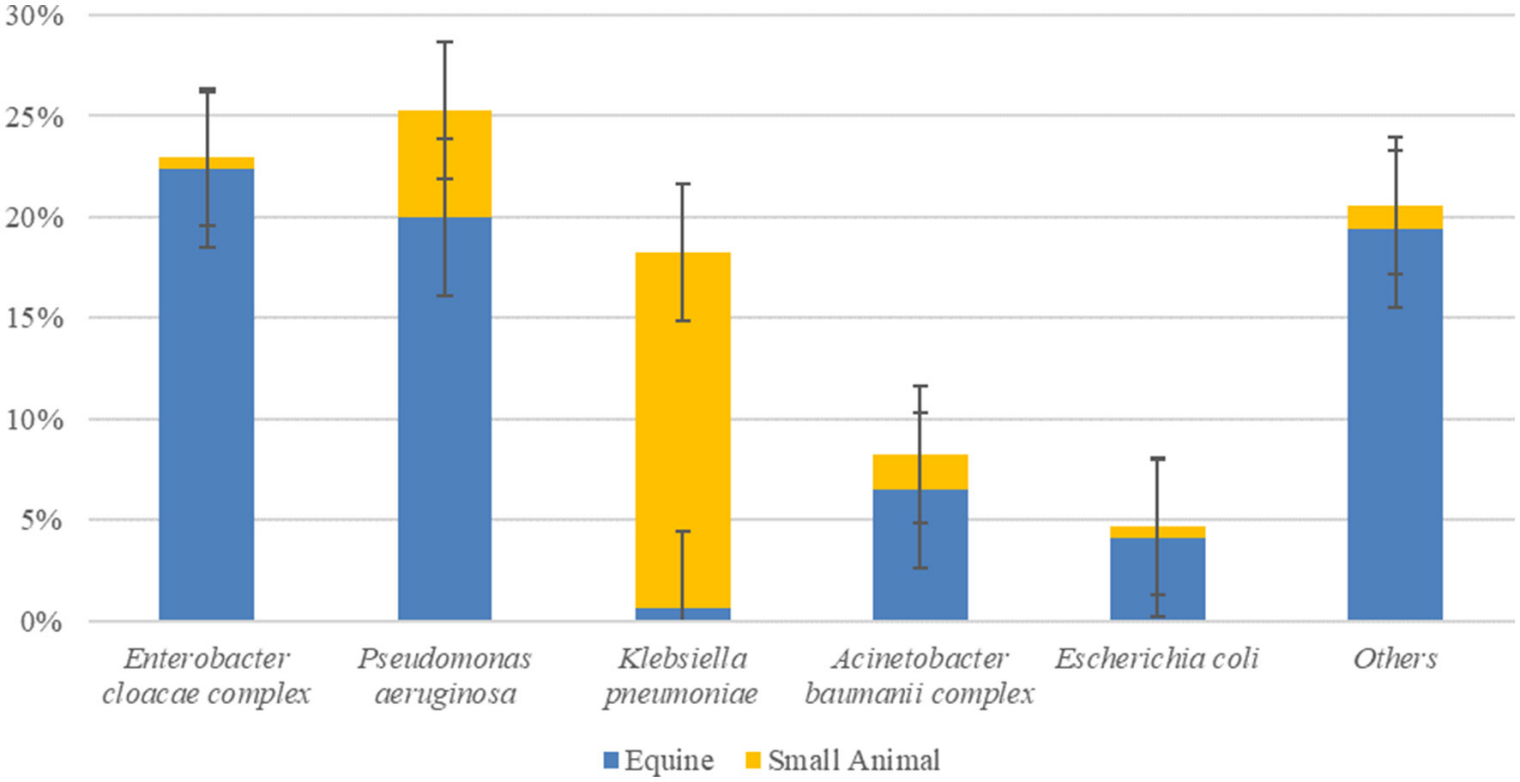
Prevalence of ESKAPE organisms, *E. coli*, and all other pathogens circulating in the equine and small animal Intensive Care Units (ICUs) between January and June 2018 during the pilot study (*n* = 170, consisting of PS-F1 and 2 and PS-ENV1 and 2 isolates). Error bars represent 95% confidence intervals.

The top ESC-R GNs isolated from the ICUs during the pilot study (January–June 2018) corresponded to the same organisms showing the highest prevalence across both hospitals during the entire study period (March 2016–June 2018). The prevalence rates of the five most commonly isolated ESC-R organisms and that of all other bacterial isolates collectively per hospital ICU are shown in Figure 2. With the exception of *K. pneumoniae* subsp. *pneumoniae*, predominantly isolated from small animal ICU, all other ESC-R GN pathogens were largely retrieved from the equine ICU.

#### 3.1.2. Prevalence of ESC-R GNs circulating in the equine hospital

**Figure 3:**
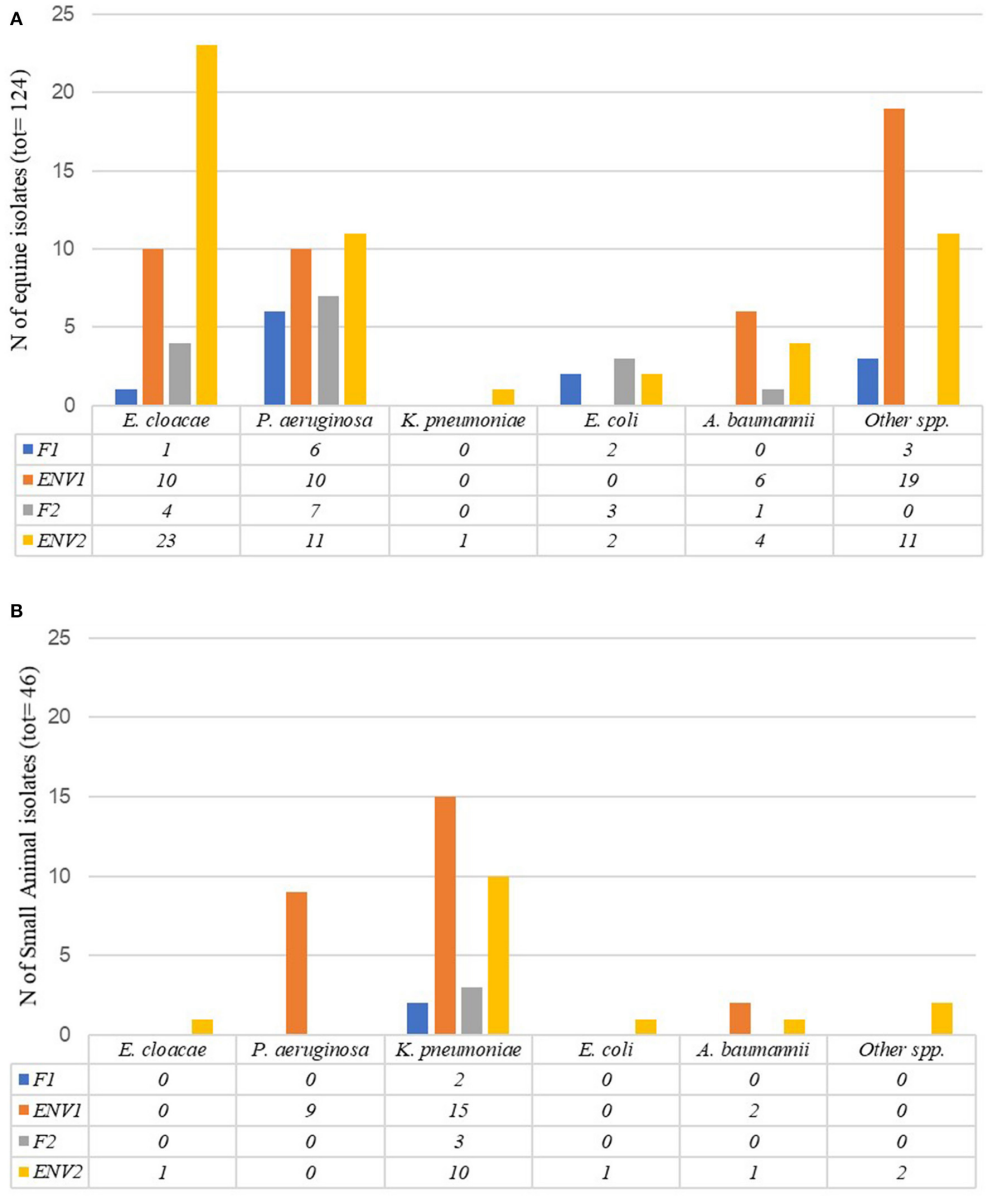
Number of ESC-R GN isolates obtained per Intensive Care Unit sample type (PS-F1 and 2 and PS-ENV1 and 2) during the pilot study (January–June 2018) in the equine **(A)** and small animal **(B)** hospital Intensive Care Units.

Within the equine ICU, 50% (100/199) of PS-F1&2 and PS-ENV1&2 samples collected between January and June 2018 yielded ESC-R GN organisms (Tables 1, 2). The distribution of ESC-R GN organisms per ICU PS sample type is shown in Figure 3A. Overall, *E. cloacae* complex and *P. aeruginosa* were most commonly recovered from all four sample types of equine origin. ESC-R *E. cloacae* complex (4/9) and *E. coli* (3/9) were cultured from equine clinical specimens during the PS.

Of the retrospective ESC-R GNs from 2016 to 2017, most represented environmental surveillance isolates (33/36) dominated by *E. cloacae* complex (Table 3). For ease, individual environmental sites detailed in Supplementary Table 2 have been grouped within unit areas from where isolated in Table 3. For example, the equine stables group comprises stable walls, floors, drains, pen window ledge and colic recovery boxes located next to the stable; the ICU unit includes keyboards, door handles, and other items such as bandage trolley or stocks located inside the ICU. Samples from non-clinical areas included students' hot desks and keyboards, in-house laboratories' worktops and equipment, washrooms, pharmacies, and reception/waiting room areas. Of all RTS environmental isolates collectively, the greatest proportion was isolated from the horse stables (18/33), followed by non-clinical areas (11/33) and ICU (4/33).

**Table 3:** Retrospective clinical (RTS-CL) and environmental surveillance (RTS-ENV) ESC-R GN isolates (March 2016–December 2017) and isolation sources within the equine and small animal hospitals.

	**Retrospective phase**			
	**Small animal hospital**		**Equine hospital**	
	**Organism species (no)**	**Site**	**Organism species (no)**	**Site**
**Clinical** **(RTS-CL)**	*E. coli* (4)	Urine	*E. coli* (1)	Skin and wound infection
*n* = 9	*P. aeruginosa* (1)	Skin and wound infection	*P. aeruginosa* (1)	Skin and wound infection
	*Salmonella* spp. (1)	Skin and wound infection	*A. baumanni* complex (1)	Skin and wound infection
**Environmental (RTS-ENV)**	*E. cloacae* complex (7)	ICU (3), non-clinical areas (2), chemotherapy (1), dermatology (1)	*E. cloacae* complex (12)	Stables (5), non-clinical areas (5), ICU (2)
*n* = 79	*P. aeruginosa* (7)	Wards (4), ICU (1), chemotherapy (1), dermatology (1)	*P. aeruginosa* (3)	Stables (1), non-clinical areas (2)
	*E. coli* (5)	Chemotherapy (1), dermatology (1), imaging (1), cardiology (1), anesthesia (1)	*E. coli* (3)	Stables
	*K. pneumoniae* (8)	ICU (3), non-clinical areas (3), dermatology (1), anesthesia (1)	*K. pneumoniae* (3)	Stables (1), non-clinical areas (2)
	*A. baumanni* complex (18)	ICU (6), non-clinical areas (7), cardiology (2), dermatology (1), wards (1), theater (1)	*A. baumanni* complex (2)	Non-clinical areas (2)
	*Serratia plymuthica* (1)	Anesthesia	*Citrobacter freundii* (4)	Stables (3), ICU (1)
			*Enterobacter aerogenes* (3)	Stables
			*Citrobacter braakii* (2)	Stables
			*Serratia marcescens* (1)	ICU

#### 3.1.3. Prevalence of ESC-R GNs circulating in the small animal hospital

Of the small animal ICU PS samples (PS-F1&2 and PS-ENV1&2, January–June 2018), 65% (52/80) were positive for ESC-R GNs (Tables 1, 2). *P. aeruginosa* was commonly detected amongst small animal ENV1 samples, whilst *K. pneumoniae* subsp. *pneumoniae* accounted for the most encountered pathogen across all four sample types in dogs at both time points. Figure 3B illustrates the distribution of ESC-R GN organisms per ICU PS sample type. The majority of PS-CL isolates detected from the small animal hospital during the same time were *E. coli* (11/24), *K. pneumoniae*, and *A. baumannii* complex (each 4/24).

Of the 52 small animal RTS GN isolates from 2016 to 2017, RTS-CL isolates were mostly *E. coli*-associated urinary tract infections (4/6). *A. baumannii* complex isolates predominated in the environment (18/46), followed by *K. pneumoniae* (8/46), *P. aeruginosa*, and *E. cloacae* complex (each 7/46) (Table 3). A broad range of units are found in the small animal hospital, of which the ICU held the highest proportion of ESC-R GNs (13/46) during the period 2016–2017.

### 3.2. Acuitas resistome test results

**Figure 4:**
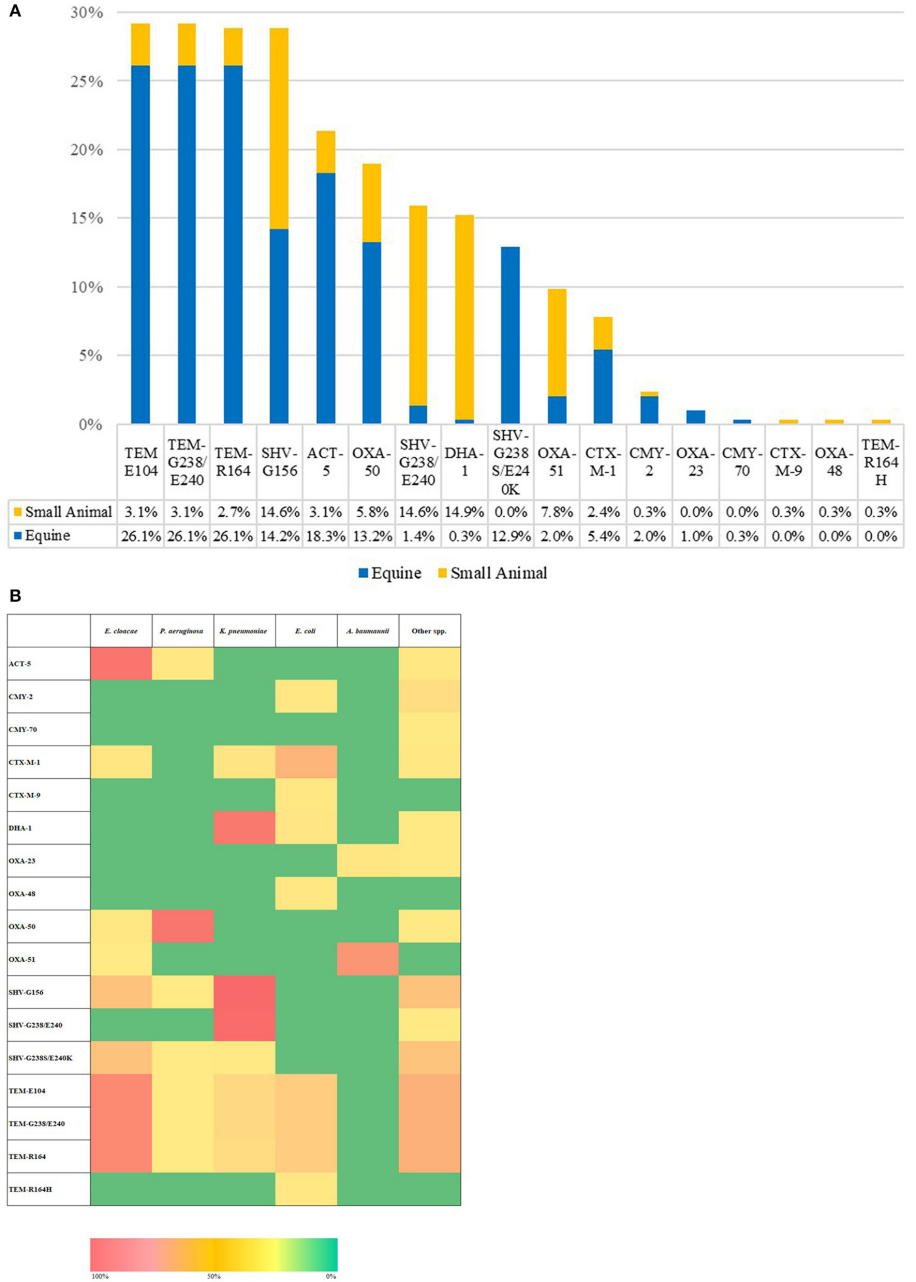
Overall extended-spectrum beta-lactamase (ESBLs), TEM/SHV/OXA, and AmpC beta-lactamase or carbapenemase genes per hospital **(A)**. Heatmap of the distribution of extended-spectrum beta-lactamase (ESBLs), TEM/SHV/OXA and AmpC beta-lactamase or carbapenemase encoding genes **(B)**; [*n* = 295, March 2016–June 2018].

Phenotypic AST results are presented within the Supplementary Figures 2, 3 and Supplementary Table 3 as this section's focus is on genotypic findings. All 295 ESC-R GNs isolates tested generated Acuitas Resistome Test results (Supplementary Table 4); overall, extended-spectrum beta-lactamase (ESBLs), TEM/SHV/OXA and AmpC beta-lactamase or carbapenemase genes were identified in 82% (242/295) of isolates whilst 18% (53/295) of ESC-R GNs had no detectable gene amongst the 67 tested belonging to 50 antibiotic resistance gene families. With regard to the overall resistome prevalence (Figure 4A), antimicrobial resistance genes harbored by ESC-R GNs varied more between hospitals than they did between PS and RTS isolates within the respective hospital. In addition, genetic makeup was frequently consistent within species of ESKAPE organisms (and *E. coli*). Figure 4B illustrates the resistome distribution across ESC-R ESKAPE and *E. coli* organisms.

Altogether, beta-lactamase enzymes of the TEM (29.2–0.3%) and SHV (28.8–12.9%) families were the most commonly detected, followed by ACT-5 beta-lactamase (21.4%). TEM and ACT-5 genes were common amongst *E. cloacae complex* isolates and, to a lesser extent, amongst *K. pneumoniae* and *E. coli*, whilst SHV enzymes were amongst *K. pneumoniae* and, secondarily, *E. cloacae complex*. Simultaneous carriage of multiple TEM and/or SHV subtypes was a feature of most positive isolates. Gene families mediating resistance to extended-spectrum cephalosporins were mostly restricted to pAmpC enzymes of the DHA (DHA-1 prevalence of 15.3%) and CMY (CMY-2 and CMY-70 prevalence of 2.4 and 0.3%, respectively) families. DHA-type enzyme was predominant amongst *K. pneumoniae* whilst CMY amongst *Citrobacter* species. CTX-M class enzymes were detected to a lesser extent than pAmpC and identified exclusively with *E. coli* CTX-M-1 (7.8%) and CTX-M-9 (0.3%). Oxacillinase enzymes of the OXA-50 (prevalence of 19%) and OXA-51 (prevalence of 9.5%) types were also detected amongst the majority of *P. aeruginosa* (53/59) and *A. baumanni complex* (27/40), respectively. One important finding is the identification of ESC- R-GN isolates carrying transmissible resistance to last-resort antimicrobials (i.e., carbapenems) within the veterinary hospitals, represented by three (1%) *Acinetobacter* spp. harboring *bla*OXA-23 and one (0.3%) *E. coli* with *bla*OXA-48. No AMR genes were identified belonging to other families (Supplementary Table 4). The proportion of ESC-R GNs carrying beta-lactamase resistance determinants amongst PS and RTS sets of isolates was 79.7% (165/207) and 87.5% (77/88), respectively.

### 3.3. Likely strain relatedness of ESC-R GNs

Genetically related types were proposed on the basis of identical bacterial species and Acuitas Resistome Test's code, whilst the Acuitas profile's AST code was used to determine subtypes. Overall, 58 Resistome types were detected across all isolates (*n* = 295; Supplementary Table 4).

**Figure 5:**
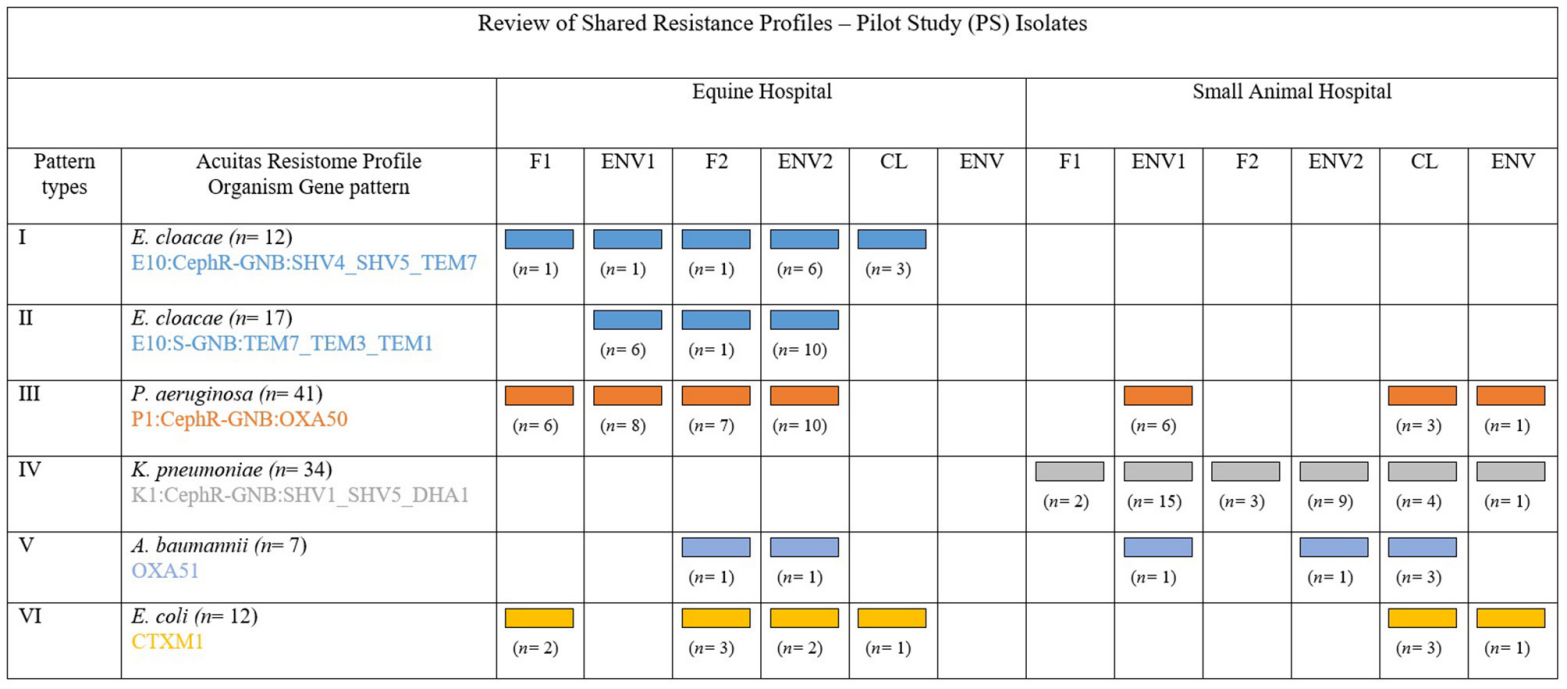
Adapted and with permission from Reuben et al. (2017). Review of the principal shared resistance profiles. Representation of five groups of MDR-GN organisms identified by Acuitas profiles as genetically related and their distribution amongst the two hospitals and sample type from where isolated during the pilot (PS). Patterns I, II, and IV show possible transmission within veterinary facilities and Patterns III, V, and VI show possible inter-facility transmission.

There was overall heterogeneity amongst profiles of typed ESC-R ESKAPE and *E. coli* organisms; nonetheless, recurrent Acuitas patterns were detected within but also across the two hospitals and especially amongst ICU pilot isolates (Figure 5). Some cluster examples of possible genetically related ESKAPE and *E. coli* pathogens are provided in the paragraphs below.

#### 3.3.1. *Enterobacter cloacae* complex

Fifty-four *E. cloacae complex* isolates from the equine hospital were grouped into nine Acuitas Resistome types and 14 subtypes (Supplementary Table 4). Of these, two major and one minor patterns were identified, consisting of 20 (Group 1: E10:CephR-GNB:SHV4_SHV5_TEM7), 19 (Group 2: E10:S-GNB:TEM7_TEM3_TEM1), and 6 (Group 3: E10:CephR-GNB:TEM7_TEM3_TEM1) isolates, respectively. Group 1 isolates harbored SHV4 & 5 and TEM7 enzymes, and this was made up of both PS (*n* = 12) and RTS (*n* = 8) isolates collected over a 2-year time period. Most PS isolates (9/12) were recovered from fecal and environmental samples connected to five horses admitted to the ICU over 24 days. *E. cloacae* complex from Group 2 carried TEM1, 3, and 7 enzymes and the vast majority (17/19) circulated during the pilot phase of the study (PS) in the equine ICU; they were associated to a total of 10 horses between March and May 2018 and were mostly found on various environmental surfaces (*n* = 14 samples) and in one fecal sample. Similarly, Group 3 consisted of PS isolates from ICU surfaces (*n* = 6) relative to four horses' pens over a month. Groups 2 and 3 isolates only differed by detection of phenotypic ESC-R in Group 3 but not in Group 2. Furthermore, all isolates from Groups 2 and 3 were of the same subtype within their respective group. In all three groups, over 50% of environmental PS isolates were collected at the second time point (ENV2 samples) and, in the case of some patients, a crossover of *E. cloacae* complex isolates from the three groups was observed in their surrounding ICU sites.

#### 3.3.2. *Pseudomonas aeruginosa*

Fifty-one out of 59 total *P. aeruginosa* isolates were typed to the same Acuitas Resistome profile P1:CephR-GNB:OXA50, consistent with OXA-50-producing organisms (Supplementary Table 4). Five subtypes were identified overall, although 44 out of 51 isolates belonged to the same AST subtype ([R70]96-[A17]181). Equine vs. small animal (35 and 16, respectively) and PS vs. RTS (41 and 10, respectively) isolates constituted the broad majority of this large *P. aeruginosa* single group. In the equine hospital, 31 *P. aeruginosa* were collected during the PS from 10 ICU patients over a 2-month period. Four horses shed this type in their feces at both time points whilst the others at either time point but predominantly with F2 samples. It was not uncommon for some horses to yield this *P. aeruginosa* type across multiple ICU samples (both fecal and environmental) with up to 80% positive samples from a single patient at both time points. At the same time, isolates of this *P. aeruginosa* type were also isolated from small animal ICU sites (*n* = 6, keyboard, kennels, floor and phone receiver) in connection to three hospitalized dogs. The majority of small animal *P. aeruginosa* of this pattern (*n* = 10) were, however, detected amongst retrospective isolates beginning in 2016. This was also the case for the equine hospital, with the first *P. aeruginosa* isolate of this type recorded in 2016.

#### 3.3.3. *Klebsiella pneumoniae*

Forty-seven *K. pneumoniae* isolates were typed in total, belonging to seven Resistome types and nine AST subtypes altogether. The vast majority (40/47) were of Resistome type K1:CephR-GNB:SHV1_SHV5_DHA1 (Supplementary Table 4); within this main group, 85% of the isolates (34/40) also shared the same AST subtype ([R70]147-[A17]180). All 40 *K. pneumoniae* were from the small animal hospital; of these, 85% were PS isolates and the remainder were RTS isolates. All but three PS isolates were linked to four dogs admitted to the ICU over a 17-day time interval in May 2018. Samples collected from these dogs displayed high K1:CephR-GNB:SHV1_SHV5_DHA1 prevalence rates at both time points (42–75% of ICU PS samples/patient). Fecal samples and multiple ICU sites (e.g., keyboard, phone receiver, door handle and individual dog kennels) were repeatedly found positive. Of note, the same *K. pneumoniae* isolate was also obtained from two clinical specimens received by the diagnostic laboratory during the same 17-day period, namely one abdominal fluid sample and one catheter-urine sample from dogs in post-surgical recovery. The peritonitis case was a patient admitted to the ICU and enrolled in the PS who yielded fecal *K. pneumoniae* at both sampling points. Other *K. pneumoniae* Resistome types carrying various combinations of SHV, TEM, and/or CTX-M-1 enzymes were identified in low numbers and random fashion.

#### 3.3.4. *Acinetobacter baumannii* complex

Forty *A. baumannii complex* isolates fell into an overall of four types and 11 subtypes with a major group containing 26 isolates and two subtypes (Supplementary Table 4). The main Resistome type A4:CephR-GNB:OXA51 consisted of OXA-51-producing *A. baumannii* identified in both hospitals (*n* = *21* small animal and *n* = 5 equine), largely being RTS isolates (*n* = 19). OXA-51 small animal isolates were cultured from a variety of hospital sites over time (including the ICU, non-clinical areas, and hospitals' departments) and from fewer clinical specimens (*n* = 3). Eleven ESC-R *A. baumanni* isolates tested negative for all AMR genes with differences in phenotypic codes reported as ESC-S, ESC-R, MDR, or CR *A. baumannii*. Importantly, Two OXA-23-producing PS isolates were identified from the water bucket and feed bowl of one horse; both were recovered at the first sampling point without repeated isolation at the second sampling. These isolates retained *in vitro* susceptibility to a number of agents, including ampicillin/sulbactam, piperacillin/tazobactam, ceftazidime, ciprofloxacin, levofloxacin, gentamicin, tobramycin, imipenem, and meropenem. A single OXA-23-producing *Acinetobacter radioresistens* isolate was obtained from another horse's hay rack in the ICU 3 days after the OXA-23-positive *A. baumannii*.

#### 3.3.5. *Escherichia coli*

ESC-R *E. coli* showed a proportionally higher degree of genetic heterogenicity when compared to the other organisms, with 36 isolates falling into eight Resistome types and 24 AST subtypes (Supplementary Table 4). One main *E. coli* group was identified (*n* = 15, Resistome profile E1:CephR-GNB:CTXM1) that contained CTX-M-1 and was further divided into eight AST subtypes; 10/15 isolates were equine and the 5/15 small animal. A small ICU cluster of seven (*n* = 7) equine PS isolates was identified from five ICU patients over 37 days. Four horses eliminated CTX-M-1-producing *E. coli* in their feces (at the second [*n* = 2], first or both [each *n* = 1] time points), and 2/5 had positive ICU surroundings. No epidemiological correlation was identified between small animal CTX-M-1-positive *E. coli* (*n* = 5, mostly PS-CL) of this Resistome type. The second largest *E. coli* group shared a lack of any AMR gene tested. Scattered *E. coli* isolates harboring combinations of TEM, CTX-M, and other beta-lactamase enzymes were identified; of note, one OXA-48 positive RTS isolate co-harboring CTX-M-9 was cultured from a dog surgical wound. This isolate retained *in vitro* susceptibility only to aminoglycosides, carbapenems and tigecycline.

## 4. Discussion

To the best of our knowledge, this is the first study to identify ESKAPE pathogens as the most prevalent ESC-R gram-negative bacteria circulating within veterinary ICUs and other hospital environments. Rapid identification of potential contamination reservoirs and understanding of the transmission dynamics of these pathogens are key to a successful infection control programme and prevention of HCAIs in both human and veterinary hospitals. Several technological advances have been implemented to guide patient management and support antimicrobial stewardship and infection control programmes in human healthcare settings; however, these are slow to be adopted within veterinary facilities.

Our study highlighted an overall high ESC-R GNs prevalence within the veterinary ICU (50% of equine and 65% of canine colonization and environmental samples altogether) and hospital environments. ESKAPE pathogens were more prevalent than the fecal contamination biomarker *E. coli* bacteria (Poirel et al., 2018), a common colonizer encountered in other veterinary hospital studies (Rubin and Pitout, 2014; Walther et al., 2014; Zogg et al., 2018). *E. cloacae* complex, *P. aeruginosa*, and *A. baumanni* complex followed by *E. coli* were more common in the equine, whilst *K. pneumoniae* predominated in the small animal hospital environment. Animal-associated gram-negative ESKAPE organisms have been reported in clinical infections (Singh, 2018) and environmental contamination of slaughterhouses (Savin et al., 2020), although no study focussed on companion animal clinics. Reports of some ESKAPE bacteria associated with companion animal ICUs and veterinary HCAIs are on the rise in recent times, particularly for MDR *A. baumannii* (van der Kolk, 2015) but are limited for other ESKAPE GN species. *Klebsiella* spp. have been described in pet nosocomial infections (Seliškar et al., 2007; Haenni et al., 2012; Ewers et al., 2014); however, considerably less data are available for *E. cloacae* (Gibson et al., 2008; Wilberger et al., 2012) and *P. aeruginosa* (Bernal-Rosas et al., 2015; Hassan et al., 2021; Soonthornsit et al., 2023) veterinary hospital dissemination and HCAIs occurrence.

Various contaminated environmental sites were identified at our hospitals (March 2016–June 2018) including ICUs (door handles, keyboards, floor, phone receivers, etc.), wards/stables (walls, floor, windows, pump holders, etc.), and non-clinical areas' high-contact surfaces (student keyboards, washroom tables, reception, etc.) amongst others. This is relevant because an increased risk of HCAIs in human patients has been demonstrated when hospital surface surroundings are contaminated (Weber et al., 2013; Nutman et al., 2016). In addition, key nosocomial pathogens have been shown to persist in the hospital environment for variable lengths of time (from days to months) acting as reservoirs of infection leading to further contamination, via staff hands or patient-to-patient transmission (Kramer et al., 2006).

Although the definition of the transmission patterns (introduction, transmission, and/or persistence) of ESC-R organisms isolated from our hospitals appears difficult, generally higher ESKAPE prevalence rates were recorded for ICU pilot samples collected at the second time point than at the first. A few possible Acuitas^®^ Resistome transmission events were recognized through the pilot study via the identification of a common Resistome profile in multiple sampling points. These included (i) *E. cloacae* complex Group 1 (E10:CephR-GNB:SHV4_SHV5_TEM7) and Group 2 (E10:S-GNB:TEM7_TEM3_TEM1) in the equine ICU; (ii) *K. pneumoniae* (K1:CephR-GNB:SHV1_SHV5_DHA1) in the canine ICU; and (iii) *E. coli* CTX-M-1 in the equine ICU, all of which circulated over a relatively short time including environmental, colonization, and clinical isolates. Furthermore, *E. coli, P. aeruginosa*, and *A. baumanii* main patterns occurred in both hospitals during the study period, suggesting possible inter-hospital spread. Staff and students may conceivably mediate cross-contamination via hands or footwear between large and small animal university hospitals located nearby (Singaravelu et al., 2023). Not infrequently, however, isolates with shared Acuitas patterns were collected at broad time intervals (months or years), also pointing toward possible pathogen persistence within hospital environments and/or to limited typing resolution.

The overall resistome trends identified TEM, SHV, and ACT-type enzymes as the most prevalent amongst companion animal ESC-R GNs in our hospitals, with lower molecular detection of ESBL (CTX-M-1 and−9), pAmpC (DHA-1, CMY-2 and−70) type enzymes and carbapenemases (OXA-23 and−48). Concerningly, the occurrence of carbapenem-resistance in critically important human gram-negative bacteria has been acknowledged in companion animals although yet at low prevalence (Rincón-Real and Suárez-Alfonso, 2022). Resistant organisms are known to spread amongst companion animals and staff in veterinary healthcare settings (Boerlin et al., 2001), and this may soon be the inauspicious case also for carbapenem-resistant bacteria, since their detection in veterinary hospital environments has been described, ranging from pet carriage to hospital outbreak (Gentilini et al., 2018; Nigg et al., 2019; Lavigne et al., 2021; Cole et al., 2022). Therefore, the implementation of routine hospital screening appears crucial to improve the surveillance of these unexpected phenotypes in veterinary settings.

The Acuitas^®^ Resistome has been employed in human hospitals for the rapid determination of carbapenemase-producing organisms (CPOs) prevalence in colonized and infected patients, for hospital and regional surveillance (Reuben et al., 2017; Lapp et al., 2021), and for the rapid information on empiric antimicrobial use (Evans et al., 2019). Its routine diagnostic applications have been trialed for fast CPO detection with promising results (Vanstone et al., 2018; Voulgari et al., 2020). Previous evaluation of the Acuitas^®^ Resistome analytical performance highlighted several benefits of this molecular approach, including simultaneous detection of a wide range of carbapenemase types with the ability to distinguish between different genotypes, the high diagnostic accuracy, the rapid turnaround time (24 h) from laboratory receipt, and high (87–100%) agreement rates with phenotypic AST (Vanstone et al., 2018; Walker et al., 2019; Voulgari et al., 2020). Limitations described include the inability to detect novel AMR genotypes and resistance to newer βlactam/inhibitor combinations in isolates of *P. aeruginosa* (Evans et al., 2019).

In our study, this novel bacterial typing method was investigated as a potential tool for conducting routine veterinary infection control and hospital surveillance as a possible alternative to conventional typing methods. Importantly, typing nosocomial MDR pathogens in “real time” has the potential to improve the cost–benefit relationship for surveillance and infection control measures through early identification and swift implementation of control procedures. The comprehensive isolate characterization offered by the Acuitas^®^ Resistome test provides an effective tool for guiding antimicrobial selection and aid in patient management, as shown in human hospitals for MDR and carbapenem-resistant *Enterobacterales, P. aeruginosa*, and *A. baumannii* (Reuben et al., 2017; Evans et al., 2019; Voulgari et al., 2020). To the best of our knowledge, this is the first application of this test to veterinary infection control; our results using veterinary ESC-R GNs suggest that this technology has great potential to provide full bacterial pheno- and genotyping in a very short timeframe. It would be beneficial to compare our findings to those obtained from larger veterinary hospital populations as we acknowledge sample size (i.e., number of patients) is a limitation of the present study. However, one limitation of this tool applied in the veterinary setting may lie in the fact that it is designed to accurately cluster clonally related pathogens carrying multiple resistance genes (Lin et al., 2015; Walker et al., 2019; Voulgari et al., 2020). Therefore, this technique may lack resolution for the detection of clonal dissemination of pathogenic bacteria harboring a reduced arsenal of AMR genes, such as OXA-50 and OXA-51-producing *P. aeruginosa* and *A. baumannii*, respectively. Also, dissemination of bacterial clones which may be virulent but not associated with known resistance genes remains undetected (Petrova et al., 2019). Furthermore, this technology is currently not cost-effective or available to perform on-site in the routine diagnostic laboratory; testing is only performed at centralized facilities in the United States and, despite the rapid test turnaround times (as early as 24 h from sample receipt) and easy online access to results in real time, there may be the delay in results generation due to sample shipping.

In conclusion, we report a high prevalence of ESC-R GN organisms, particularly of the ESKAPE group of pathogens, amongst clinical, colonization and environmental samples collected at two UK veterinary hospitals with emphasis on their ICUs (equine and small animal). This included the detection of resistance to last-resort antimicrobials (i.e., carbapenems) carried by four isolates. The Acuitas^®^ Resistome test is a useful technology for veterinary infection control purposes, allowing to track intra-hospital dissemination of specific genotypes as suspected here for *E. cloacae* and *K. pneumoniae* in the equine and small animal hospital ICUs, respectively. Possible inter-hospital spread of certain ESKAPE genotypes was also detected, which may be consistent with staff or student movement across hospitals. Nevertheless, further typing is necessary to confirm the spread of genetic types, especially for those carrying comparably less AMR genes than their human nosocomial counterparts; for this reason, the Acuitas^®^ Resistome test may be more beneficial in human rather veterinary hospital settings at present. Further research is warranted to investigate the occurrence and molecular epidemiology of ESKAPE GNs within veterinary hospitals and the correlations these have with veterinary HCAIs, as such pathogens may be more widespread in veterinary settings than currently acknowledged, in similar but less alarming trends than seen in human hospitals.

## Data availability statement

The original contributions presented in the study are included in the article/Supplementary material, further inquiries can be directed to the corresponding author.

## Ethics statement

The animal studies were approved by University of Liverpool Veterinary Research Ethics Committee. The studies were conducted in accordance with the local legislation and institutional requirements. Written informed consent was obtained from the owners for the participation of their animals in this study.

## Author contributions

CI and JD supported sample collection in the equine hospital, whilst VS and RR in the small animal hospital. FZ processed the hospital samples and wrote the manuscript together with DT. All authors read and approved the manuscript for submission.

## Footnotes

^1^Healthcare-associated Infections Surveillance Network (HAI-Net). *European Centre for Disease Prevention and Control*. Available online at: https://www.ecdc.europa.eu/en/about-us/partnerships-and-networks/disease-and-laboratory-networks/hai-net (accessed January 11, 2022).
