# Supplementary material for: Resistome-based surveillance identifies ESKAPE pathogens as the predominant gram-negative organisms circulating in veterinary hospitals
Source: Front Microbiol. 2023 Sep 7;14:1252216. doi: 10.3389/fmicb.2023.1252216 (PMC10513425; doi:10.3389/fmicb.2023.1252216)
Supplement: Supplementary file 1 [file Data_Sheet_1.zip › Supplementary Materials Folder/Supplementary_Materials and Captions.docx]

Supplementary Material

Resistome – Based Surveillance Identifies ESKAPE Pathogens as the Predominant Gram-Negative Organisms Circulating in Veterinary Hospitals

**Flavia Zendri^1^, Cajsa M. Isgren^2^, Jane Devaney^2^, Vanessa Schmidt^1,3^, Rachel Rankin^,3^, Dorina Timofte^1*^**

*** Correspondence:** Dorina Timofte [d.timofte@liv.ac.uk](mailto:d.timofte@liv.ac.uk)

# Supplementary Data

Supplementary Material includes sections detailing the antimicrobial susceptibility testing (AST) results. This is provided separately owing to focusing the main body of the manuscript on molecular typing as opposed to phenotypic findings.

Additionally, comprehensive sample and typing result tables are comprised in this material.

*Antimicrobial susceptibility testing (AST) results*

*Overall ESC-R Gram-negative organisms AST*

AST was conducted on a total of 286 ESC-R GNs owing to failure in identifying nine of the 295 isolates. Depending on the antibiotic/pathogen combinations, between 3.1% and 39.2% of the isolates had no interpretative criteria available for certain antibiotics, expressing either intrinsic resistance mechanisms (as with *E. cloacae complex* and other *Enterobacterales* against ampicillin/sulbactam, *P. aeruginosa* against ertapenem or *Ochrobactrum anthropi* against beta-lactams) or due to the lack of established interpretative criteria (the case of some environmental opportunistic bacterial genera and species as *Pantoea* spp. or *Stenotrophomonas maltophilia*), which in both cases resulted in a proportion of the isolates’ AST results not being reported (NR = Not Reported). In addition, AST results could not be determined for a small proportion of the isolates (0.3% to 2.1%) against some antibiotics (ND = Not Determined). Overall, ESC-R GNs displayed highest susceptibility rates towards carbapenems (100% for both imipenem and meropenem whilst two isolates [*Serratia* and *Cronobacter* spp.] were non-susceptible to ertapenem), followed by aminoglycosides (81% susceptibility to amikacin, 64% to tobramycin and 63% to gentamicin), tigecycline (73% of susceptible isolates) and quinolones (approximately 65% of isolates susceptible to both ciprofloxacin and levofloxacin). Non-susceptibility rates were highest for aminopenicillins, monobactams (18% of isolates susceptible to both ampicillin/sulbactam and aztreonam) and cephalosporins (ranging from only 6% of isolates susceptible to cefazolin to 55% susceptible to cefepime) as well as trimethoprim/sulfamethoxazole (38% of susceptible isolates) (Figure 5). Because most ESC-R GNs were obtained through screening on selective media inhibiting growth of ESC-S isolates, resistance to cephalosporins was expected; nonetheless, the vast majority of *E. cloacae* and *P. aeruginosa* ICU isolates were susceptible to cefepime (100% and 97%, respectively) as did *P. aeruginosa* and *A. baumannii* complex to ceftazidime (96% and 90%, respectively). On the contrary, *K. pneumoniae* and *E. coli* isolates’ susceptibility rates were low (6% of *K. pneumoniae* and 33% of *E. coli*) to the same antibiotics. No phenotypic resistance to carbapenems was detected among the collection of companion animal ESC-R ESKAPE plus *E. coli* isolates.

*AST of ESC-R GNs circulating in the equine hospital*

One-hundred-sixty (*n*= 160) ESC-R GNs out of 169 PS and RTS equine isolates generated AST results, shown in Table 6. Of the antimicrobial compounds commonly employed in clinical veterinary practice, *P. aeruginosa* isolates (*n*= 38) retained vast susceptibility to the quinolones (95% for levofloxacin to 97% for ciprofloxacin), aminoglycosides (92% for gentamicin to 100% for amikacin), cefepime and ceftazidime (both 95%). *E. cloacae* complex (*n*= 54) isolates’ susceptibility rates were lowest for aminoglycosides (11%) except amikacin (100%) and for trimethoprim-sulfamethoxazole (13%), intermediate for the quinolones (61% for ciprofloxacin to 65% for levofloxacin) and ceftriaxone (54%) and highest for cefepime (100%). Equine *E. coli* isolates (*n*= 14) were largely non-susceptible to most antimicrobial classes aside from carbapenems, with the exception of amikacin, tigecycline and piperacillin-tazobactam (100% susceptible to all) whilst *A. baumannii* complex (*n*= 15) retained broader susceptibility, to potentiated aminopenicillins (87% for ampicillin/sulbactam), quinolones (87% for ciprofloxacin and 80% for levofloxacin) and aminoglycosides (87% for gentamicin and tobramycin).

As the ESKAPE pathogens (and *E. coli*) isolated during the PS from the equine ICU (January-June 2018) accounted for a significant proportion (115/160) of the overall equine ESC-R GNs through the whole study (March 2016 – June 2018), comparable AST trends to those described overall were observed amongst ICU isolates alone (Figure 6a). Worthy of note, two ESC-R GN from the equine ICU PS tested resistant towards ertapenem, consisting of one *Serratia fonticola* and one *Cronobacter sakazakii* group isolated from the environment surrounding two hospitalised horses.

When comparing PS and RTS equine isolates however, *E. cloacae* complex isolates (the most prevalent equine ESC-R GN organism across both groups of isolates) from the RTS phase appeared generally less susceptible than PS-*E. cloacae* complex to ESC and monobactams, quinolones and aminoglycosides except amikacin. RTS-*P. aeruginosa* from the equine hospital were largely non-susceptible to gentamicin whereas ICU PS-*P. aeruginosa* retained 100% susceptibility to this antibiotic; this was observed to a much lesser extent also for 4^th^ generation cephalosporins and levofloxacin (but not ciprofloxacin).

*AST of ESC-R GNs circulating in the small animal hospital*

All PS and RTS small animal ESC-R GN isolates (*n*= 126) were subject to AST, and results are shown in Table 6. Likewise, the findings in the equine hospital, 100% of small animal *P. aeruginosa* (*n*= 21) were susceptible to quinolones, aminoglycosides, cefepime and ceftazidime. Of the *K. pneumoniae* isolates (*n*= 43), all were non-susceptible to 3^rd^ and 4^th^ generation cephalosporins and most to the quinolones (only 2% susceptible to levofloxacin and 5% to ciprofloxacin). On the other hand, substantial susceptibility was retained against trimethoprim-sulfamethoxazole (84%) and aminoglycosides (100% for amikacin and gentamicin and 86% for tobramycin). Small animal *E. coli* (*n*= 22) were generally susceptible to aminoglycosides (100%, 91% and 86% for amikacin, tobramycin and gentamicin, respectively), moderately susceptible to quinolones (73%), piperacillin-tazobactam (64%) and trimethoprim-sulfamethoxazole (59%) and lesser susceptible to 3^rd^ and 4^th^ generation cephalosporins (41%). All (100%) *A. baumannii* complex (*n*= 25) isolates tested susceptible against ampicillin/sulbactam, quinolones and aminoglycosides (gentamicin and tobramycin) and most to trimethoprim-sulfamethoxazole (96%) and ceftazidime (92%). AST findings for the small subset of *E. cloacae* isolates (*n*= 10) from this hospital were partially aligned to those from equine: however, susceptibility rates were much higher for aminoglycosides (100%), trimethoprim-sulfamethoxazole (90%) and the quinolones (90% and 80% for ciprofloxacin and levofloxacin, respectively).

AST results of *K. pneumoniae* (*n*= 30) and *P. aeruginosa* (*n*= 9), the predominant ESKAPE pathogens (and *E. coli*) isolated from the small animal ICU during the PS (January-June 2018), were also vastly in accordance with the overall figures for the same bacterial species isolated throughout the study period (March 2016 – June 2018), particularly for *P. aeruginosa*. Slightly higher resistance rates were seen among ICU *K. pneumoniae* compared to overall *K. pneumoniae* resistance trends for the quinolones (none susceptible to levofloxacin and 3% to ciprofloxacin from the ICU) and piperacillin/tazobactam (none susceptible from the ICU versus 2% overall) whereas higher susceptibility rates were detected among ICU isolates compared to overall figures for tigecycline (100% susceptible from the ICU versus 91% overall), tobramycin and trimethoprim-sulfamethoxazole (each 100% susceptible from the ICU). AST profiles of these two ESKAPE pathogens were largely identical among small animal ICU GN isolates of the same bacterial species (i.e. most or all *K. pneumoniae* and *P. aeruginosa* isolates were either susceptible or resistant to the same antimicrobial). Upon comparison of such ICU profiles with those of the same ESKAPE organisms isolated from PS-CL and PS-ENV samples (consisting of five and four *K. pneumoniae* and *P. aeruginosa* isolates, respectively), striking overlap was observed also among isolates within this larger subset obtained between January and June 2018 from ICU and non-ICU patients and hospital sites. The same was observed for ICU and non-ICU *A. baumannii* PS isolates (of which three ICU and four PS-CL) but not for *E. coli (*of which two ICU and 11 PS-CL).

Almost identical AST profiles were also shared between *K. pneumoniae*, *P. aeruginosa* and *A. baumannii* PS and RTS isolates obtained throughout the study period.

# Supplementary Figures and Tables

## Supplementary Figures

**Supplementary Figure 1:** The Acuitas Lighthouse Profile consists of five sections combining to form a unique fingerprint for an organism; the sections are Organism ID code: Phenotype code: Top 3 Resistome Test Genes code: Resistome Profile code: AST Profile code. Downloaded from the Acuitas Lighthouse MDRO Management System (OpGen) website portal.

**Supplementary Figure 2**: Overall antimicrobial susceptibility testing findings based on phenotypic screening of 286 pilot (PS) and retrospective (RTS) ESC-R GN organisms belonging to 23 bacterial species isolated across both Hospitals (March 2016 – June 2018).

**Supplementary Figure 3**: Antimicrobial susceptibility data attributable to the pilot isolates (PS-F1&2 and ENV1&2) from the equine (2a) and small animal (2b) ICUs (January-June 2018). The number of isolates per bacterial species or group of bacteria is indicated in the legend in brackets. Percentages express the susceptibility rates against the tested antibiotics, excluding pathogen-antibiotic combinations not illustrated owing to the lack of interpretative criteria (“Not Reported”); NR: *E. cloacae complex*-ampicillin/sulbactam, *P. aeruginosa*-aztreonam, *P. aeruginosa*-ertapenem, *A. baumannii complex*-amikacin, *A. baumannii complex*-cefepime, *A. baumannii complex*-ertapenem.

## Supplementary Tables

## Supplementary Table 1: Patients, samples and ESC-R GN isolates obtained from faecal (F) and environmental (ENV) surrounding specimens of horses and dogs admitted to the hospital ICUs during the Pilot Study (PS, January-June 2018). F1 & ENV1= faecal and environmental samples collected upon ICU admission; F2 & ENV2= faecal and environmental samples collected after 48 hours from ICU admission; EMBA: Eosin Methylene Blue Agar; ICU: Intensive Care Unit.

**Supplementary Table 2:** Clinical and environmental equine and canine ESC-R GN isolates collected prior (RTS-CL & RTS-ENV, March 2016-December 2017) and during the pilot study (PS-CL & PS-ENV, January-June 2018) that were included in the analysis. SSIs: surgical site infection swabs. A, C & D are designated units within the equine hospital.

**Supplementary Table 3:** Overall antimicrobial susceptibility testing (AST) results of pilot study (PS) and retrospective (RTS) ESC-R GN companion animal ESKAPE + *E. coli* isolates included in the study (March 2016 – June 2018). The percentage of non-susceptible (resistant - R + intermediate - I) isolates (*n*= 286) per antibiotic^a^ per hospital is shown.

**Supplementary Table 4:** Overall Acuitas® Resistome test typing results of ESC-R GN organisms (n= 295) isolated from the equine and small animal hospital (March 2016- June 2018). PLEH: Philip Leverhulme Equine Hospital; SATH: Small Animal Teaching Hospital; F1 & ENV1= faecal and environmental samples collected upon ICU admission; F2 & ENV2= faecal and environmental samples collected after 48 hours from ICU admission; CL= clinical sample; ENV= environmental sample from the wider hospital environments.

**
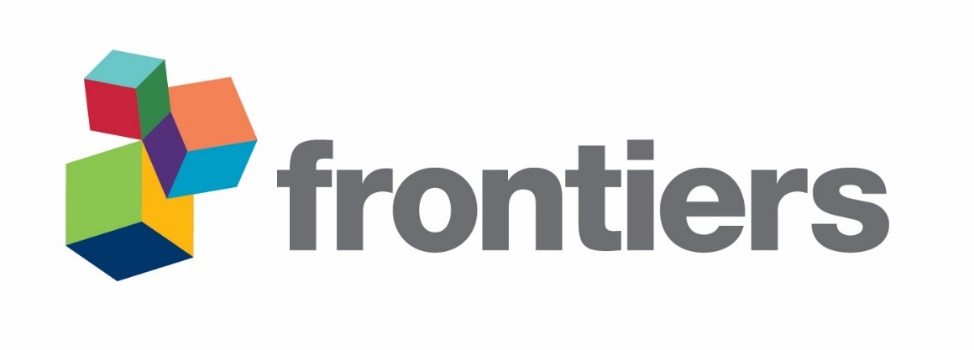
**
