# Supplementary figures and images for: Resistome-based surveillance identifies ESKAPE pathogens as the predominant gram-negative organisms circulating in veterinary hospitals
Source: Front Microbiol. 2023 Sep 7;14:1252216. doi: 10.3389/fmicb.2023.1252216 (PMC10513425; doi:10.3389/fmicb.2023.1252216)

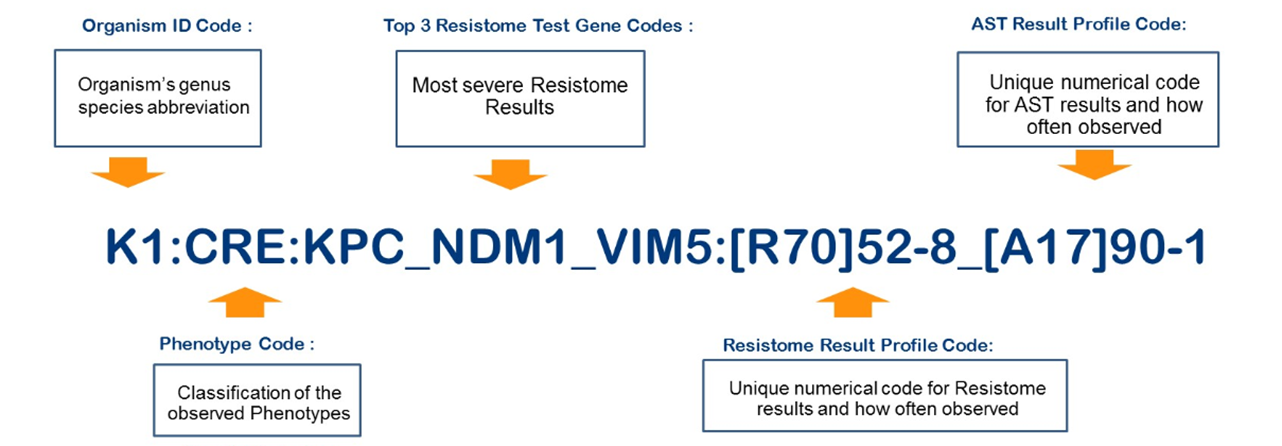

Supplement: Supplementary file 1 [file Data_Sheet_1.zip › Supplementary Materials Folder/Supplementary Figure 1.jpg]

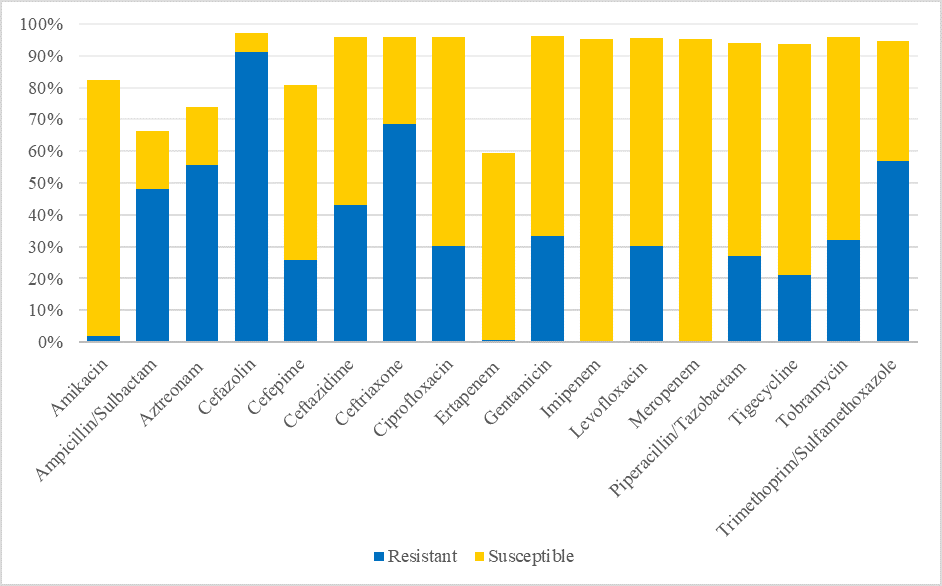

Supplement: Supplementary file 1 [file Data_Sheet_1.zip › Supplementary Materials Folder/Supplementary Figure 2.jpg]

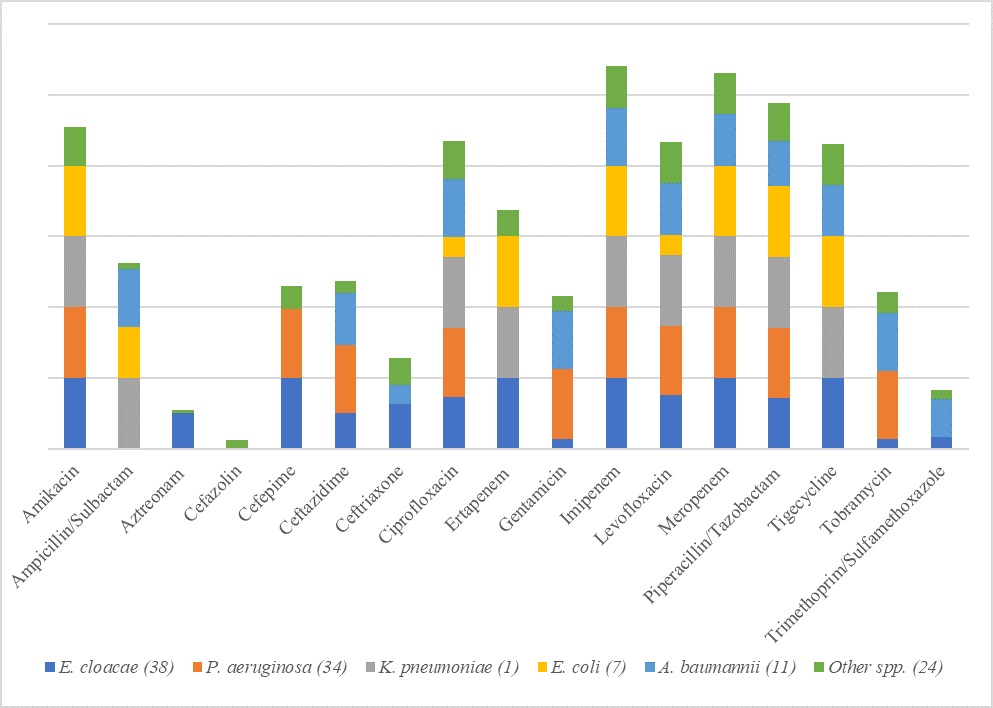

Supplement: Supplementary file 1 [file Data_Sheet_1.zip › Supplementary Materials Folder/Supplementary Figure 3a.jpg]

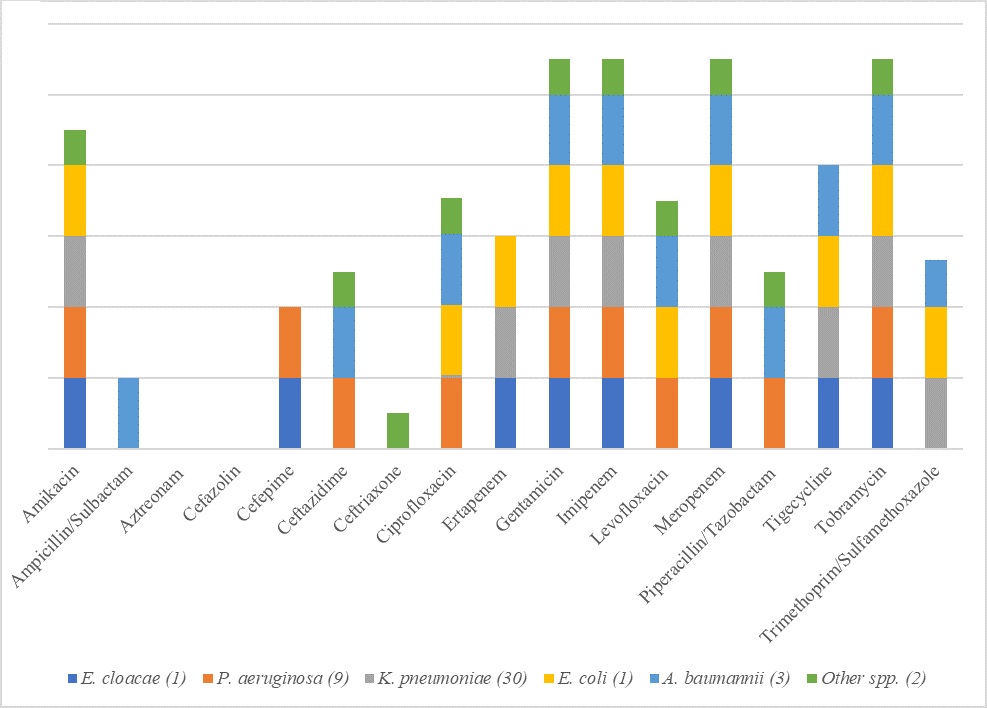

Supplement: Supplementary file 1 [file Data_Sheet_1.zip › Supplementary Materials Folder/Supplementary Figure 3b.jpg]
